# Neuronal Plasticity in the Entorhinal Cortex

**DOI:** 10.1155/2008/314785

**Published:** 2008-12-31

**Authors:** C. Andrew Chapman, Roland S. G. Jones, Min Jung

**Affiliations:** ^1^Center for Studies in Behavioral Neurobiology, Department of Psychology, Concordia University, Montreal, QC, Canada H4B 1R6; ^2^Neuronal Networks Research Group, Department of Pharmacy and Pharmacology, University of Bath, Bath BA2 7AY, UK; ^3^Neuroscience Laboratory, Institute for Medical Sciences, Ajou University School of Medicine, Suwon 443-721, South Korea

The entorhinal cortex (EC) is a unique and fascinating structure, constituting a highly parallel interface between phylogenetically old cortex (hippocampus) and higher neocortical areas. The literature related to the EC has increased dramatically in the past 30 years. A simple PubMed search for EC lists around 20–40 articles per year in the early 1980s. This more than doubled by the beginning of the 1990s and doubled again by the start of the new century. Throughout the current decade, some 220–250 papers per year relating to structure, function, and pathology of the EC have been published, and the numbers are still rising. It has become increasingly apparent that the EC can no longer be regarded as a simple relay between the hippocampus and neocortex, but is a dynamic processor of both efferent and afferent information. In recent years, there has been burgeoning interest in synaptic plasticity at entorhinal synapses particularly in relationship with learning and memory functions of limbic structures. Neuroplasticity in the EC clearly takes many forms. In addition to the “input” and “output” roles that have long been ascribed to the superficial and deep layers, functions of the entorhinal area have been studied with respect to differences in the medial and lateral divisions, prominent state-dependent theta- and gamma-frequency population activities, membrane conductances that shape cellular and network activities, the neurophysiology of synaptic inputs from other regions and interlaminar connections, and the powerful roles of modulatory transmitter systems and local inhibition. The discovery of grid cells in the EC has led to strong interest in the role of the area in spatial processing. On the other side of the coin, long-term adaptive changes in EC function also appear to play pivotal roles in neuropathological states, particularly epilepsy, schizophrenia, and Alzheimer's disease.

Contributions to this special issue of Neural Plasticity provide an overview of some of the theoretical and methodological approaches that are being applied to understand the functions and mechanisms of neuroplasticity within the EC. The research contained in this special issue deals mainly with findings derived from animal research. It is clear, however, that research using a variety of techniques is providing insight into entorhinal function in human and clinical populations.

The significance of plasticity in the EC is best understood within the context provided by anatomical knowledge of the extrinsic and intrinsic connectivity of this area. Canto et al. have provided an excellent overview of the anatomical organization of the EC. Their analysis of intrinsic and extrinsic connections, in relationship to laminar organization and neuronal subtypes, heavily underscores our increasing perception of the EC as a dynamic interactive processor essential to hippocampal function, rather than a passive route of information entry and exit. The EC is tightly intertwined with circuitries of the hippocampal formation and other cortical areas, and its particular contributions to sensory processing, learning, memory, and motor behavior have been difficult to define. Lipton and Eichenbaum present evidence dealing with the complementary roles of the EC and hippocampus in episodic memory. They have found that medial EC neurons show stronger trajectory-dependent firing whereas hippocampal place cells show greater spatial specificity. Based on these observations, they propose roles of the hippocampus and medial EC in encoding sequences of events and disambiguating overlapping experiences, respectively. A contribution by Bevilaqua et al. also highlights the role of the EC in extinction learning and how this is affected by ageing. They raise the possibility that ageing-associated impairment of extinction may occur in part because of degenerative changes in the EC.

Several articles in the special issue have investigated mechanisms of long-term, activity-dependent synaptic plasticity. Ma et al. observed differences in the induction of long-term synaptic potentiation (LTP) in the superficial neurones of the EC; LTP in horizontal (potentially extrinsic) inputs required NMDA receptor activation whereas LTP in interlaminar (intrinsic) inputs did not. This underlines the potential complexity of information processing in neurones that provides the bulk of hippocampal afferent inputs. Hernández et al. have found that prenatal malnutrition has lasting effects on the capacity of the EC to express LTP, and it is possible that this reduced plasticity may contribute to deficits in learning and memory in the adult. In addition to LTP, there is a growing body of literature dealing with characteristics of long-term *depression* (LTD) in the EC. Kourrich et al. have investigated the postsynaptic signaling mechanisms that mediate entorhinal LTD, and provide further evidence for the roles of calcium-dependent signaling and protein phosphatases in the expression of LTD in the superficial layers. Much of the interest in long-term changes in synaptic strength has traditionally been driven by interest in mnemonic processes and neurological disorders. Short-term activity-dependent changes in synaptic strength also have powerful influences on ongoing synaptic transmission in the EC. In this issue, Chamberlain et al. have continued their work on the role of presynaptic NMDA autoreceptors in excitatory transmission in layer V of the EC. They demonstrate that presynaptic NR2B subunits are critical in enhancement of both spontaneous glutamate release and frequency-dependent facilitation of action potential-driven release. The kinetics of these receptors provide an optimal facilitation frequency of 3–6 Hz, and the authors speculate on the possible involvement of the autoreceptors in generation of delta/theta oscillations in mnemonic processing and epileptogenesis.

The EC is subject to the powerful influence of several neuromodulatory transmitters including acetylcholine, serotonin, and dopamine. One of the articles in the special issue, from Caruana and Chapman, describes the dose-dependent, bidirectional effect of dopamine on the amplitude of evoked synaptic responses in layer II of the lateral EC. The significance of this bidirectional effect for entorhinal function is as yet unclear, but there are interesting parallels with bidirectional effects of dopamine in prefrontal cortex.

Oscillatory neuronal population activities are known to contribute to the synchronization of neuronal activity in cortical areas throughout the brain, and the mechanisms that generate theta- and gamma-frequency activities in the EC are being examined. Kainic acid induces powerful gamma oscillations in layer III of the EC, and Stanger et al. demonstrate here that the GLU_K5_ subunit is required for kainate-induced gamma activity in the EC. Morgan et al. also examined kainate-induce oscillations in the beta/gamma range in EC with respect to the action of cannabinoid receptors. Their experiments suggest that CB1 receptors are constitutively active in the EC and that antagonists or inverse agonists enhanced beta/gamma oscillations in layer II but suppressed oscillations in layer V, again pointing to differential control of synchrony in neurons providing afferent input to the hippocampus and those receiving output from it. Hasselmo and Brandon have examined how oscillations in membrane potential in entorhinal neurons, combined with the unique persistent firing activity in these cells, may contribute to several phenomena. This provides an excellent example of how quantitative analysis of cellular and network properties of the EC can lead to a greater understanding of mechanisms of cognition and behavior. The role of oscillatory activity in regulating large-scale interactions between the hippocampal formation and the neocortex has come under increased study in the past several years as several labs have begun to examine concurrent changes in oscillatory EEG activity in hippocampus and neocortex associated with sleep and waking states. In the current issue, Axmacher et al. describe results obtained from scalp and intracranial EEG recordings from epilepsy patients that show increased correlations between oscillations in the neocortex and medial temporal lobe including the rhinal cortex during non-REM sleep; the increased correlation may support mechanisms related to memory consolidation.

Our hope is that this special issue of Neural Plasticity will serve to emphasize the diversity of phenomena related to neural plasticity in the EC, and to highlight the importance of these effects, particularly in relationship to the increasing perception of the participation of the EC in mnemonic function of temporal lobe memory circuits and its related roles in integration of spatial information.

*C. Andrew Chapman* *C. Andrew Chapman* *Roland S. G. Jones* *Roland S. G. Jones* *Min Jung* *Min Jung*

